# Syntactic and semantic specialization in 9- to 10-year-old children during auditory sentence processing

**DOI:** 10.1038/s41598-024-76907-8

**Published:** 2024-11-06

**Authors:** Jin Wang, Neelima Wagley, Mabel Rice, Nadine Gaab, James R. Booth

**Affiliations:** 1 School of Education and Information Studies, University of California, Los Angeles, CA USA; 2 Speech and Hearing Sciences, Arizona State University, Tempe, AZ USA; 3 Child Language Doctoral Program, University of Kansas, Lawrence, KS USA; 4 Graduate School of Education, Harvard University, Cambridge, MA USA; 5 Department of Psychology and Human Development, Vanderbilt University, Nashville, TN USA

**Keywords:** Semantics, Syntax, Sentence processing, Developmental, Multi-voxel pattern analysis, Human behaviour, Language

## Abstract

Prior literature has debated whether syntax is separable from semantics in the brain. Using functional magnetic resonance imaging and multi-voxel pattern analysis, our previous studies investigated brain activity during morpho-syntactic versus semantic processing. These studies only detected semantic specialization in activation patterns and no syntactic specialization in 5- to 6-year-old and 7- to 8-year-old children. To examine if older children who have mastered morpho-syntactic skills would show specialization for syntax, the current study examined 64 9- to 10-year-old children using the same design and analyses. We observed that only the left IFG pars opercularis was sensitive to syntactic but not semantic information, supporting the hypothesis that this region serves as a core region for syntax. In addition, the left STG which has been implicated in the integration of semantics and syntax, as well as the left MTG and IFG pars triangularis which have been implicated in semantics, were sensitive to both semantic and syntactic information with no evidence of specialization. These findings suggest a lexicalized view of syntax, which argues that semantically sensitive regions are also critical regions for syntactic processing during language comprehension.

## Introduction

Children are born with brain cortices that have broad functionality. With development, some cortical regions become selectively responsive to a certain type of stimuli or tasks but not others^1–3^. This developmental change from broad to narrower functionality is referred to as functional specialization^4^. Neuro-developmental disabilities are often characterized as having delayed processes of specialization or atypical patterns of specialization^5,6^. Therefore, understanding the development of functional specialization in typically developing children not only contributes to our knowledge of brain development, but also provides a reference to determine atypical development in children with developmental disabilities.

In the language domain, prior neuroimaging studies have consistently shown that adult brains are specialized for phonological and semantic processing during tasks that require processing single words. Specifically, the left superior temporal gyrus (STG) and the dorsal inferior frontal gyrus (IFG) tend to show greater activity for phonological than semantic processing, whereas the left middle temporal gyrus (MTG) and the ventral IFG tend to exhibit greater activity for semantic than phonological processing^7–11^. In our previous neuroimaging studies, we observed double dissociation evidence in children showing that phonological and semantic specialization is evident in the temporal lobe at 5- to 6-years old^12^, progresses to the frontal lobe at 7- to 8-years old^13^, and shows near adult-like patterns at 9- to 10-years-old^14^. These findings suggest a developmental increase in language specialization, consistent with the Interactive Specialization account^4^. The findings also support the neurocognitive model of language comprehension proposed by Skeide and Friederici^15^, which argues for a specific developmental trajectory with the initial development of bottom-up processing in the temporal lobe followed by top-down processing in the frontal lobe.

While phonological and semantic specialization during word-level processing and its developmental transition from the temporal to the frontal lobes have been demonstrated in developing children, little consensus has been reached regarding semantic and syntactic specialization during sentence-level processing. For example, in Friederici’s^16^ language comprehension model, the left IFG pars opercularis is thought to be specialized for syntactic processing. In addition, the left IFG triangularis and MTG are assumed to be specialized for semantic processing, and the left STG is thought to be involved in integrating syntactic information from the left IFG pars opercularis and lexical information from the left MTG. In the memory, unification, and control (MUC) model proposed by Hagoort^17^, however, the left IFG pars opercularis and STG are associated with phonological processing. The left IFG pars triangularis and MTG are thought to be specialized for syntactic processing, and the left IFG orbitalis and inferior temporal gyrus (ITG) are related to semantic processing. Different from these two language models^16,17^ which hypothesize distinct brain regions for semantic and syntactic processing, Fedorenko et al.^18^ argues that syntactic processing is not separable from lexico-semantic processing. They did not observe any language-related brain regions, identified by their localizers, that were more engaged in syntactic than lexico-semantic processing, although some regions showed the reversed pattern. Matchin and Hickok^19^ proposed a lexicalized view of syntax, in which they suggest that the IFG triangularis and MTG are two core areas for processing both semantic and syntactic information during speech comprehension and/or production. Furthermore, Matchin^20^ argues that it is difficult to cleanly dissociate syntax from semantics as lexical items host syntactic information (e.g., lexical category). Any modification to lexico-semantic content in experimental stimuli will also assuredly tax syntactic processing. The difficulty of separating syntax from semantics is a problem for localizationist models. However, the development of syntax-semantics may be grounded in other network configurations. In summary, the field is still debating where syntactic processing occurs in the brain and whether it is separable from semantic processing. Regardless of the debates among various language theories, the core regions related to auditory sentence processing are relatively consistent, involving the left IFG, STG, and MTG.

In terms of the neural development of semantic and syntactic specialization, only a few studies in children have directly compared semantic and syntactic tasks or conditions to examine brain specialization during auditory sentence processing. Brauer and Friederici^21^ examined German-speaking 5- to 6-year-old children but only observed that the left IFG pars opercularis showed greater activation for the syntactically violated sentences than the semantically violated and/or correct sentences. No brain regions showed greater activation for the semantically violated sentences than the syntactically violated and/or correct sentences. Wu et al.^22^ studied German-speaking 5- to 6-year-olds using a sentence-picture matching task with a design of 2 syntactic complexity (subject- vs. object-initial sentences) by 3 semantic manipulations (animacy hierarchies: animate subject + inanimate object, vs. animate subject + animate object, vs. animate subject + inanimate object sentences). However, they only found a main effect of semantic manipulations in the left IFG pars triangularis but no main effect of syntactic manipulations. These single dissociation results^21,22^ do not support language specialization because it could be simply due to one manipulation being more difficult than the other. Skeide et al.^23^ used a sentence-picture matching task with a design of 2 syntactic complexity (subject- vs. object-initial sentences) by 2 plausibility (plausible vs. implausible sentences). They examined German-speaking children in three age groups (i.e., 3–4, 6–7, and 9–10 years old). They found that children gradually separated the semantic and syntactic processes in distinct brain regions, with only 9- to 10-year-old children showing a syntactic main effect in the left IFG pars opercularis. This study suggests a developmental progression of increased brain specialization for semantic and syntactic processing, supporting the Interactive Specialization account^4^. However, it is the only developmental study showing a double dissociation and therefore, more work is needed to understand how semantic and syntactic specialization changes over development. Besides, Skeide et al.^23^ only used word orders (i.e., subject- or object-initial sentences) as their syntactic manipulation. Whether morpho-syntax, a critical syntactic skill gradually acquired with development and often used as an indicator for identifying specific language disorder^24^, also shows syntactic specialization remains unclear. Although Matchin^20^ pointed out that semantics and syntax are often intertwined in a sentence, manipulating morpho-syntactic markers will affect the meaning of a sentence to a lesser extent than manipulating word order. This is because, according to the optional infinitive account of language acquisition^25^, morpho-syntax is more rule-based, and even typically developing children misuse morpho-syntactic markers (e.g., finiteness markers such as -s, -ed, DO, or BE) until about age 9. Therefore, studying morpho-syntax provides a valuable alternative to help us understand the neural specialization of syntax and semantics in developing children.

To fill the literature gap in developmental research, our previous studies investigated syntactic and semantic specialization using a grammaticality judgement task that taps into morpho-syntactic processing, and a plausibility judgement task that taps into semantic processing, in children younger than 9 years old. In our study on 5- to 6-year-old children, we observed that the left MTG was specialized for semantic processing whereas the left STG was sensitive to both semantic and syntactic information with no specialization for either^26^. We further observed that those effects remained in 7- to 8-year-old children. In addition, the left IFG pars triangularis started to show semantic specialization during incorrect sentence processing^27^. In both studies, semantic specialization was only observed using multi-voxel pattern analyses (MVPA)^28^ but not univariate analyses. Together, the two studies suggest a progression of semantic specialization from the temporal to frontal lobe during auditory sentence processing. This observation is consistent with previous studies on word-level processing^12,13^ and aligns with the neurocognitive model of language development from temporal to frontal regions as proposed by Skeide and Friederici^15^. However, neither study on auditory sentence processing observed syntactic specialization in the brain. As reviewed above, there is still debate about whether syntax is separable from semantic processing in adult brains^16,18^, and the previous developmental research^23^ did not report syntactic specialization in the left IFG pars opercularis until children were 9- to 10-years old. The lack of a syntactic effect in children ages 5 to 8 in our previous studies^26,27^ could be due to the younger age of the participants or because syntactic processing is inseparable from semantics as argued by Fedorenko et al.^18^. We acknowledge that the lack of syntactic specialization observed in our previous studies could also be attributed to the insensitivity of fMRI measures in capturing the appropriate resolution of syntactic neural activity. Other, more precise approaches, such as single-cell intracranial recording, may be able to detect these subtleties. However, this is beyond the scope of the current study.

The current study investigated if morpho-syntactic specialization is present in 9- to 10-year-old children by using the same experimental design and applying both univariate and MVPA analyses as in our previous studies^26,27^. Behavioral studies show that 9–10 years old is when children have mostly mastered morpho-syntactic knowledge^24^. According to the developmental neuroimaging findings by Skeide et al.^23^ and our previous studies on younger children^26,27^, we hypothesized that 9- to 10-year-old children would continue to show semantic specialization in the left MTG and IFG pars triangularis, and sensitivity to both types of information with no specialization in the left STG. Additionally, they may exhibit syntactic specialization in the left IFG pars opercularis. This would be consistent with Interactive Specialization theory^4^ which argues for increased specialization as children develop. All hypotheses and analytical procedures were pre-registered https://osf.io/bf3au.

## Methods

### Participants

Data used in this study were pulled from a publicly available dataset (see data descriptor in Wang et al.^29^, and dataset at https://openneuro.org/datasets/ds003604/versions/1.0.7). All participants invited to attend the functional magnetic neuroimaging (fMRI) sessions had no prior diagnoses of attention deficit hyperactivity disorder (ADHD), neurological diseases, psychiatric disorders, learning disabilities or language disorders, or hearing loss. All participants had normal or corrected-to-normal vision as reported by their guardians in the exclusionary survey. The experimental procedure was approved by the Institutional Review Board at University of Texas at Austin.

Participants were asked to complete several screening assessments, which included 5-handedness questions in which the child had to pretend to write, erase, pick-up, open, and throw something, and the Diagnostic Evaluation of Language Variation (DELV) Part I^30^. Children also completed standardized assessments measuring non-verbal IQ and language skills. Non-verbal IQ was measured using the Matrices subtest of the Kaufman Brief Intelligence Test, Second Edition (KBIT-2)^31^. General language skill was measured using the Core Language Score of the Clinical Evaluation of Language Fundamentals, Fifth Edition (CELF-5)^32^.

One hundred and one participants completed at least one of fMRI tasks. Participants included in the current study met the following six criteria: (1) Complete data for both runs of the grammaticality and plausibility tasks (15 excluded); (2) Primarily right-handed, defined as performing at least 3 out of 5 items using their right hand during the handedness assessment (0 excluded); (3) Mainstream English speakers, as categorized by the Part I Language Variation Status sub-test on the DELV (4 excluded); (4) IQ and language skills with a standardized score of higher than 70 for both the KBIT-2 non-verbal subtest (4 excluded) and the CELF-5 Core Language Score (0 excluded); (5) Acceptable accuracies for each run during the in-scanner tasks (see more details in the experimental procedure section, 9 excluded); (6) Acceptable head movement during the in-scanner tasks, defined as participants having no more than 10% or 6 consecutive outlier volumes in each run (see more details in the preprocessing section, 5 excluded). Sixty-four participants (36 girls, mean age 9.20 ± 0.19, range 9.0–9.9 years old) who passed the screening criteria were included in the final analysis.

### Experimental procedure

#### Stimuli

All sentence stimuli in the grammaticality task and the plausibility task had the following structure: An optional carrier phrase (“Last week”/“Every day”) + subject and verb phrase (e.g., “She baked”) + number and object (e.g., “two cakes”). The sentences included one of the following four verb forms: (1) Third person present tense (-s); (2) Present progressive copula (be); (3) Auxiliary verb (do); and (4) Simple past tense (-ed). Each condition had five sentence stimuli for each verb form (see below for a description of conditions). Stimuli were matched across all conditions in each task in terms of the written word frequency^33,34^, the number used (one /two /three /four /five /six), the subject used (he/she/they), the number of syllables (6–8), and the frequency of “not” usage in the sentences. The auditory sentences were recorded in a sound insulated booth by using Audacity software. All sentences were read by one female native English speaker who was asked to briefly pause between phrases. All sentences were then segmented into two (subject phrase + object phrase) or three (i.e., carrier phrase + subject phrase + object phrase) sections. Consistent pauses (approximately 500 ms) were added in between phrases using Praat software so that all sentences were similar in their pacing.

#### Grammaticality task

In each trial, children heard one auditory sentence, presented binaurally through earphones. There were three conditions of sentence stimuli: grammatically correct (Gram), finiteness violation (FVio), and plurality violation (Pvio) (examples, see Table 1). A carefully matched frequency-modulated white noise burst served as the auditory perceptual control (PC) condition. The children were asked, “does the way she speaks sound right?” They were instructed to respond to all trials as quickly and accurately as possible, using their right index finger for a yes response in the Gram condition, and using their right middle finger for a no response in Pvio and Fvio conditions. Children were asked to press the yes button with their right index finger whenever they heard the PC condition. Throughout the trial, a blue circle remained on the screen during the auditory stimuli presentation and turned yellow 1000ms before the trial ended to remind the participants to respond. The duration of each sentence was 2700ms to 4500ms. The duration of the response interval was 2300 ms. To optimize the extraction of the hemodynamic response, inter-trial intervals of 0, 575, or 1150 ms were added randomly in equal proportions, resulting in a duration of 5000 ms to 7950 ms for each trial. The length of trials was equated across conditions. The four conditions were pseudo-randomized so that there were no more than 5 same responses in a row. There were 20 trials for each condition, totaling 80 trials evenly divided into two runs. Each run lasted around 4.5 min.

**Table 1:** Examples of the grammaticality task and the plausibility task.

Task	Condition	Response	Brief Explanation	Example
Grammaticality task	Gram	Yes	Grammatical	Every day, they play one game
	FVio	No	Finiteness violation	He dropping one book
	PVio	No	Plurality violation	She is fixing two clock
	PC	Yes	Perceptual control	“Shh – Shh”
Plausibility task	SCon	Yes	Strongly congruent	Last week, she baked two cakes
	WCon	Yes	Weakly congruent	He does not break two glasses
	InCon	No	Incongruent	They are bouncing one paper
	PC	Yes	Perceptual control	“Shh – Shh”

The three sentence conditions in the grammaticality task were designed according to the following standards. The plurality violation condition was defined as the mismatch between the number and object by either adding an “s” or omitting an “s” in the object noun word. The finiteness violation condition was defined as the inconsistency between the subject and verb phrase by either adding an inflection or omitting an inflection/auxiliary verb. The grammatically correct condition was defined as sentences without grammatical errors.

#### Plausibility task

In each trial, children heard one auditory sentence, presented binaurally through earphones. There were three conditions of the sentence stimuli: strongly congruent (SCon), weakly congruent (WCon) and incongruent (InCon) (examples, see Table 1). A carefully matched frequency-modulated white noise burst served as the auditory perceptual control (PC) condition. The children were asked, “does the way she speaks make sense?” They were instructed to respond to all trials as quickly and accurately as possible by using the right index finger for a yes response in SCon and WCon conditions and using the right middle finger for a no response in the InCon condition. Children were asked to press the yes button with their right index finger whenever they heard the PC condition. The presentation procedure was the same as the grammaticality task. There were 20 trials for each condition, totaling 80 trials evenly distributed in two runs. Each run lasted approximately 4.5 min.

The three sentence conditions in the plausibility task were designed according to the following standards. The two congruent conditions were based on the association strength values between the verb and the object as defined in the University of South Florida Free Association Norms^35^. The strongly congruent condition had an association of 0.28–0.81 (M = 0.41, SD = 0.12) between the verb and the object in the sentence. The weakly congruent condition had an association of 0.02–0.19 (M = 0.11, SD = 0.05) between the verb and the object in the sentence. In the incongruent condition, the verb and the object in the sentence had no semantic association.

#### Scanning procedure, exclusionary criteria, and contrasts selection

Prior to taking part in the fMRI scanning session, participants were required to complete a mock scan session. They performed the same task in the mock scanner to ensure that they understood the task and were acclimated to the scanner environment. Different stimuli were used in the mock and real scanning sessions. The real scanning took place within a month of the practice session.

Acceptable in-scanner performance was defined as greater than 50% accuracy on the perceptual control and the ‘easy’ (i.e., SCon and PVio) conditions. No evidence of response bias was defined by an accuracy difference no greater than 40% between the InCon (requiring a ‘no’ response) and SCon (requiring a ‘yes’ response) conditions in the Plausibility task and between the PVio (requiring a ’no’ response) and Gram (requiring a ‘yes’ response) conditions in the Grammaticality task.

Because the grammaticality and plausibility tasks shared the structure of sentence stimuli and only differed in their emphasis on syntactic or semantic processing induced by task requirements, comparing brain activity from the two tasks allowed us to investigate the question of syntactic and semantic specialization in developing children. Like the contrasts used in our previously studies on 5- to 6-year-old and 7- to 8-year-old children^26,27^, the Gram and the SCon conditions were chosen as the best contrast to explore the semantic and syntactic specialization. This is because on the one hand, both conditions required the same response (pressing the ‘yes’ button), excluding the possible confounding factor that different responses might induce different brain activation patterns; on the other hand, both conditions were correct sentences, avoiding the potential confusion in processing anomalous sentences^36,37^. The strongly congruent (SCon) condition rather than the weakly congruent (WCon) condition was used because the former was more natural and semantically predictable. In the exploratory analyses of our previous study on 7- to 8-year-old children^27^, we also analyzed incorrect sentences and observed that semantic specialization in the frontal lobe only appeared during incorrect but not correct sentence processing. This observed frontal effect may be due to more linguistic unification and cognitive control needed for processing incorrect sentences^17^. Although incorrect sentences may also induce more domain-general processes such as error detection or executive function, our comparison between the two tasks within incorrect sentences likely canceled out the shared phonological or domain-general cognitive processes. As a result, the observed effects of task comparisons for incorrect sentences predominately reflected our targeted language processes (i.e., semantics or syntax). Therefore, for the completeness of analyses, the current study also analyzed incorrect sentences and contrasted the FVio and InCon conditions to examine semantic and syntactic specialization in the brain. The finiteness violated (FVio) condition rather than the plurality violated (PVio) condition was used because the former tapped into a core morphosyntactic skill^24^, whereas the latter may rely primarily on the semantic processing of the number words. The exploratory analyses for PVio versus InCon conditions are provided in the supplementary material.

### Data analysis

#### Preprocessing

The SPM12 toolbox (Statistical Parametric Mapping; http://www.fil.ion.ucl.ac.uk/spm) was used to analyze the data. First, all functional images were realigned to their mean functional image across runs. Then, the anatomical image was segmented and warped to the pediatric tissue probability map template^38^ to obtain the transformation field. An anatomical brain mask was created by combining three segmentation products (i.e., grey, white, and cerebrospinal fluid), and then applied to its original anatomical image to produce a skull-stripped anatomical image. Next, we co-registered the mean functional image and all functional images to the skull-stripped anatomical image. All functional images were then normalized to the pediatric template by applying the transformation field. The pediatric tissue probability map template that is appropriate for our age group was created using CerebroMatic^38^, a tool that makes SPM12 compatible pediatric templates with user-defined age, gender, and magnetic field. Art-Repair (http://cibsr.stanford.edu/tools/human-brain-project/artrepair-software.html) was used to identify outlier volumes, defined as those with volume-to-volume head movement exceeding 1.5 mm in any direction, or more than 4% deviation in global mean signal intensity. The outlier volumes were interpolated by the values from adjacent good volumes and were then de-weighted from 1 to 0.01 at the first-level analyses^39^. Participants with more than 10% or more than 6 consecutive outlier volumes in each run were excluded from the analysis.

#### Regions of interest

Given most of previous language models include the left IFG, STG, and MTG^16,17,19^, four left language regions of interest were created: IFG pars opercularis, IFG pars triangularis, STG, and MTG. The four language masks of interest were defined as the overlap between functional activation map at the group level (voxel wise threshold p = 1) and anatomical mask of interest created by using the anatomical automatic labeling (AAL) atlas in the WFU PickAtlas tool (http://www.nitrc.org/projects/wfu_pickatlas). In the univariate analysis, we combined the four ROIs into one big mask to check if there were significant clusters that show either semantic or syntactic specialization within the language areas. In the multi-voxel pattern analysis, we analyzed activation patterns separately in each of the four language ROIs.

#### Univariate analysis

A canonical univariate analysis, which allows for comparison with other prior studies using univariate analyses, was conducted first. Data was smoothed with a 6-mm isotropic Gaussian kernel after normalization. Art-Repair was then used to interpolate outlier volumes after smoothing. First-level analysis was performed on smoothed data with a traditional general linear model (GLM). The onsets of each condition from both the grammaticality and the plausibility task were entered as regressors of interest. Six movement parameters estimated from the realignment step were entered as regressors of no interest and the repaired interpolated volumes were de-weighted to control for movement effects. A high pass filter with a cutoff of 128s and an SPM default artificial mask threshold of 0.5 was applied. First, we defined the simple contrasts of (Gram minus PC), (FVio minus PC), (SCon minus PC), and (InCon minus PC) to display brain engagement during the different types of sentence processing. We then defined the contrasts of (Gram minus PC) > (SCon minus PC) and (FVio minus PC) > (InCon minus PC) to calculate brain activation maps for syntactic specialization during correct and incorrect sentence processing, respectively. Similarly, we used the contrasts of (SCon minus PC) > (Gram minus PC) and (InCon minus PC) > (FVio minus PC) to calculate brain activation maps for semantic specialization during correct and incorrect sentence processing, respectively. A group-level one-sample *t*-test was conducted to find regions that were specialized for syntactic or semantic specialization within the combined language mask. In addition, we used a conjunction analysis^40^ (https://osf.io/rhzm6) to find regions that showed common activation across tasks that were sensitive to both syntactic and semantic information.

#### Multi-voxel pattern analysis

Unsmoothed data was used to perform both feature selection and multi-voxel pattern analysis. For feature selection, we first estimated a traditional GLM with each condition from both the grammaticality and plausibility tasks. Then, contrast maps for all sentence conditions versus perceptual control conditions across tasks were generated. We chose the top 250 most activated voxels for the contrast within each of the four language ROIs (i.e., the left IFG pars opercularis and triangularis, the left MTG, and the left STG) separately regardless of significance. These top 250 voxels served as the features (voxels) that were the most sensitive to auditory sentence processing for the multi-voxel pattern analysis. The overlap among participant’s individualized top 250-voxel ROI within each mask is plotted in Fig. 4.

**Fig. 1:**
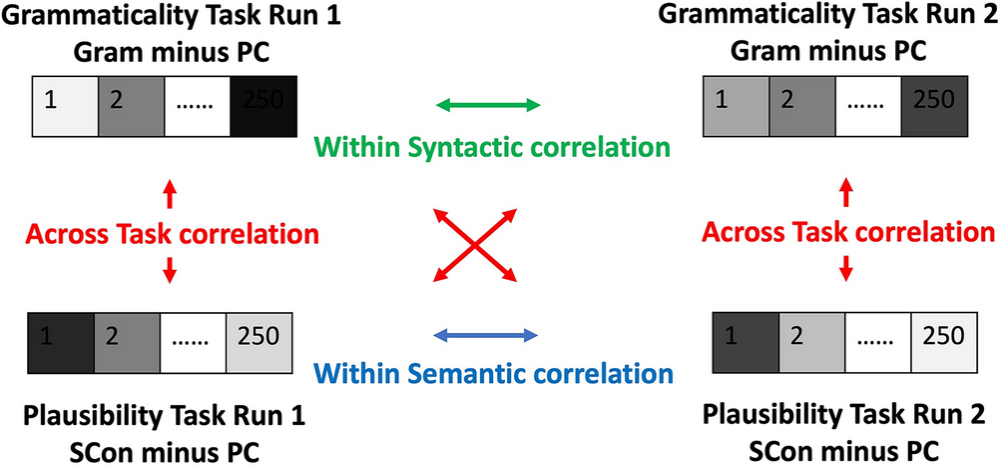
Generation of first-level results using the Haxby et al.^28^ approach of multi-voxel pattern analysis (MVPA). Here we display the analyses of correct sentences as an example. Gram: Grammatical condition; SCon: Strongly congruent condition; PC: perceptual control condition. The top 250 voxels for all sentences > perceptual control across two tasks within each ROI were used as individualized ROIs to extract brain activity features. The within-syntactic (in green) or within-semantic (in blue) correlations were calculated by correlating brain activity (t values) of the top 250 voxels between run1 and run2 within each task for each participant. Four across-task correlations (in red) between Grammaticality_Run1 and Plausibility_Run1, between Grammaticality_Run2 and Plausibility_Run1, between Grammaticality_Run2 and Plausibility_Run1, and between Grammaticality_Run2 and Plausibility_Run2 were generated and then averaged for each participant. This figure was adapted from Wang, Rice, & Booth^26^.

Using a similar approach to a correlational multi-voxel patten analysis (MVPA) proposed by Haxby et al.^28^, we compared the within-task and across-task correlations in the top 250 voxels within each ROI to examine semantic and syntactic specialization. For each task, we had two runs (i.e., run1 and run2). After estimating the GLM, for the analysis of correct sentence processing, the contrast t-maps for Gram minus PC or SCon minus PC were generated for each run. The t-values in each voxel from the top 250 voxels within each ROI were then extracted using 3dMaskDump in AFNI toolbox. The within-semantic task correlation for each participant was calculated by correlating the t-values of the top 250 voxels for SCon minus PC in the plausibility task run1 with the t-values of the top 250 voxels for SCon minus PC in the plausibility task run2. In the same way, the within-syntactic task correlation for each participant was calculated by correlating the t-values of the top 250 voxels for Gram minus PC in the grammaticality task run1 with the t-values of the top 250 voxels for Gram minus PC in the grammaticality task run2. As for the across-task correlation, the t-values of the top 250 voxels for Gram minus PC in each run of the grammaticality task were correlated with the t-values of the top 250 voxels for SCon minus PC in each run of the plausibility task, resulting in 4 between task correlations. The across-task correlation for each participant was then calculated by averaging the 4 between task correlations. Figure 1 shows the generation of first-level results using a correlational MVPA proposed by Haxby et al.^28^. After getting the within- and across-task correlations from each participant, we conducted paired sample t-tests to compare the within-semantic and within-syntactic correlations with the across-task correlations at the group level in each region to test our hypotheses. Bonferroni correction was used to determine the significance of the results.

**Fig. 2:**
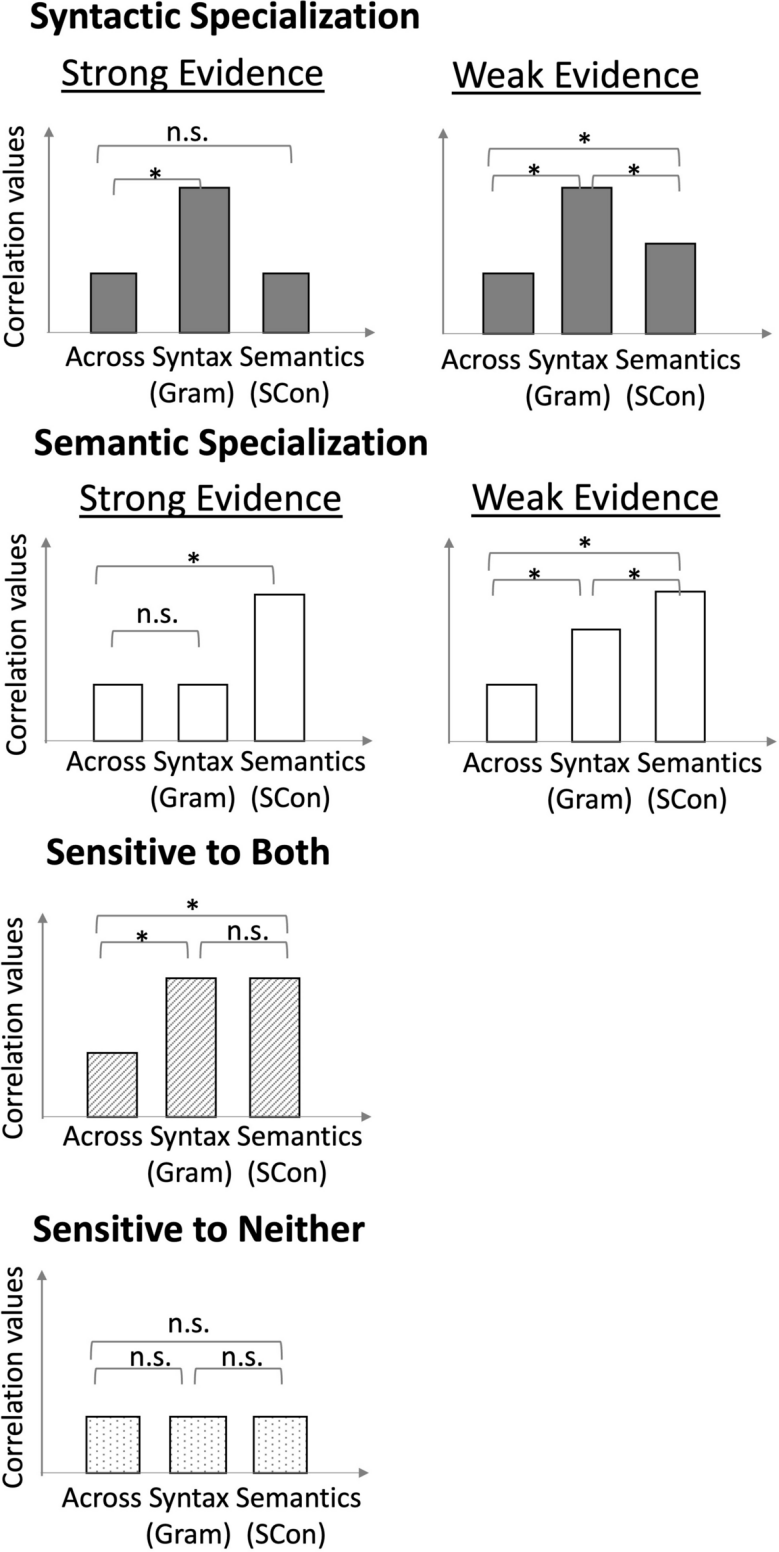
Hypotheses of different outcomes in group level analyses. Here we display the outcomes of correct sentences as an example. X axis displays the different types of correlations. Syntax (Gram): the within-syntactic correlations using grammatically correct condition; Semantics (SCon): the within-semantic correlations using strongly congruent condition; Across: across-task correlations. Y axis represents the correlation values calculated from the first level multivoxel pattern analysis. "*" indicates that the two types of correlations differ significantly. "n.s." represents no significant difference. This figure was adapted from Wang, Wagley, Rice, & Booth^27^.

There could be four potential outcomes (see Fig. 2). One outcome would be the evidence of syntactic specialization. Strong evidence would be within-syntactic correlations > across-task correlations, and within-semantic correlations = across-task correlations. Weak evidence would be within-syntactic correlations > across-task correlations, within-semantic correlations > across-task correlations, and within-syntactic correlations > within-semantic correlations, indicating that although this region is sensitive to both semantic and syntactic information, it shows preference for syntax over semantics. The second outcome would be evidence of semantic specialization. Strong evidence would be within-semantic correlations > across-task correlations, and within-syntactic correlations = across-task correlations. Weak evidence would be within-syntactic correlations > across-task correlations, within-semantic correlations > across-task correlations, but within-semantic correlations > within-syntactic correlations. The third possible outcome would be evidence of sensitivity to both semantic and syntactic information with no specialization as indicated by within-syntactic correlations > across-task correlations, within-semantic correlations > across-task correlations, and within-semantic correlations = within-syntactic correlations. The fourth possible outcome would be evidence of sensitivity to neither semantic nor syntactic information, as indicated by within-syntactic correlations = within-semantic correlations = across-task correlations.

In addition to the analyses of correct sentences using the contrasts of Gram minus PC and SCon minus PC, the same analytical procedure was applied for the contrasts of FVio minus PC and InCon minus PC for the examination of syntactic and semantic specialization during incorrect sentence processing.

## Results

### Behavioral performance

Descriptive statistics for the in-scanner task performance of the final sample (N = 64 participants) are presented in Table 2. Reaction times were calculated only based on trials in which the participant responded accurately (i.e., with the correct judgement response).

**Table 2:** Descriptive statistics for the in-scanner task performance (N = 64).

Task	Condition	Accuracy (%)		Reaction Time (ms)	
		Mean (SD)	Range	Mean (SD)	Range
Grammaticality task	Gram	87.5 (9.8)	50–100	2217 (178)	1823–2584
	FVio	72.7 (20.0)	5–100	3639 (276)	3202–4783
	PVio	90.2 (11.3)	55–100	2193 (180)	1872–2725
	PC	96.5 (4.8)	85–100	1561 (960)	518–4728
Plausibility task	SCon	90.8 (10.4)	65–100	1738 (223)	1322–2581
	WCon	82.8 (12.1)	50–100	1872 (213)	1547–2742
	InCon	91.4 (8.9)	60–100	1843 (197)	1487–2288
	PC	98.4 (3.3)	85–100	1513 (874)	589–4332

Gram: grammatical correct condition; FVio: finiteness violated condition; PVio: plurality violated condition; PC: perceptual control condition; SCon: strongly congruent condition; WCon: weakly congruent condition; InCon: incongruent condition. Inclusion criteria include: the accuracies of the PVio, SCon, and PC conditions >  = 50%, the accuracy differences between PVio and Gram < 40%, and the accuracy differences between SCon and InCon < 40%. SD = standard deviation. N = number of participants.

### Brain results of univariate analysis

**Fig. 3:**
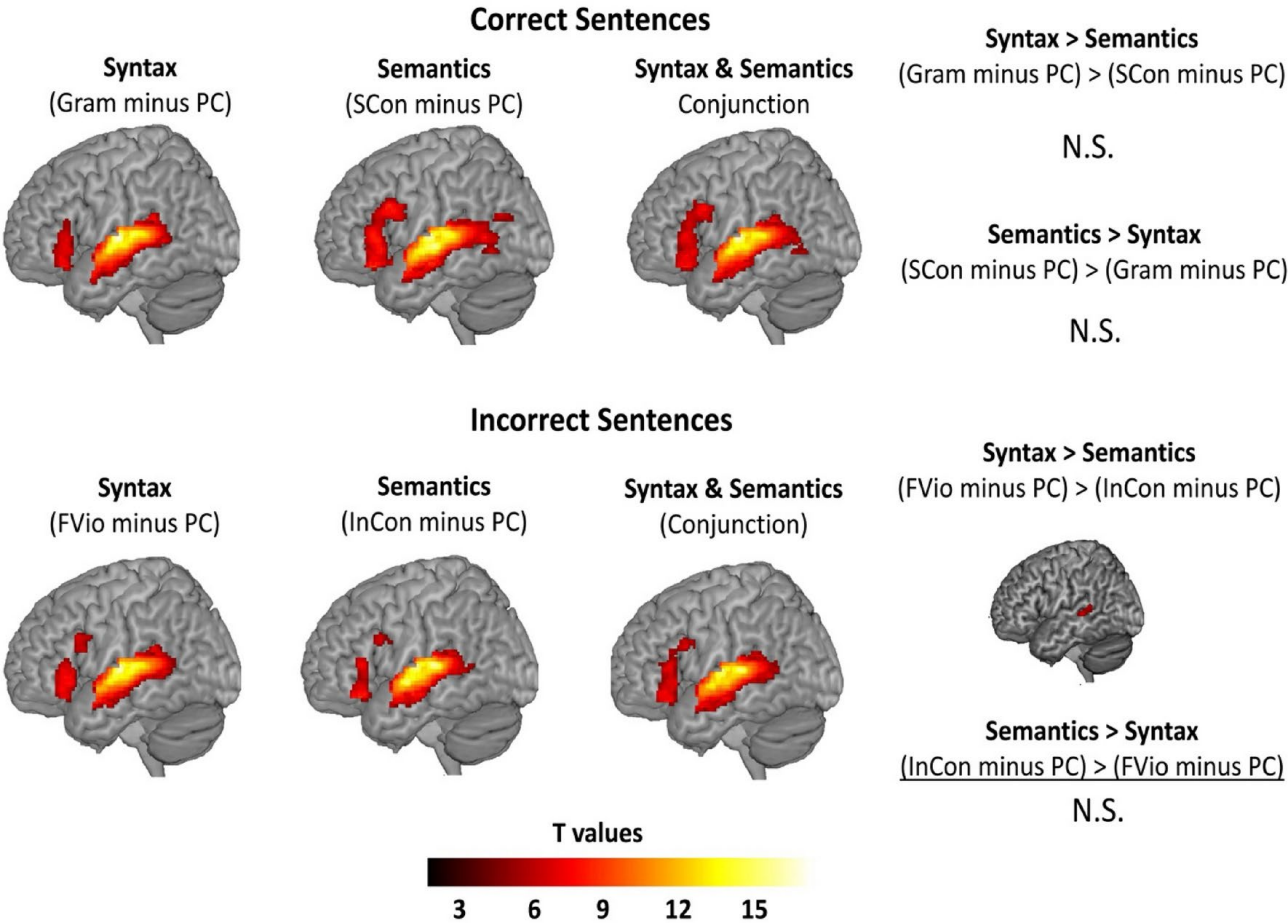
Brain activation maps with significant clusters within the left hemisphere language mask at a voxel-wise *p* < 0.001, cluster-wise *p* < 0.05 family-wise error (FWE) corrected threshold using SPM12 small-volume correction. Gram: grammatical correct condition; SCon: strongly congruent condition; FVio: finiteness violated condition; InCon: incongruent condition; PC: perceptual control condition. N.S. stands for no significant clusters observed.

We did not find any significant clusters for the contrast of (Gram minus PC) > (SCon minus PC) or (SCon minus PC) > (Gram minus PC) during correct sentence processing. Conjunction analysis showed that the two tasks largely overlapped with each other in the left STG, MTG, and the left IFG pars triangularis and opercularis. In addition, we did not find any significant clusters for the contrast of (InCon minus PC) > (FVio minus PC) during incorrect sentence processing. However, we did find a significant cluster in the left superior temporal sulcus (STS) for the contrast of (FVio minus PC) > (InCon minus PC). Again, conjunction analysis showed that the two tasks largely overlapped with each other across the language areas in the left STG, MTG, and the left IFG pars triangularis and opercularis. All brain activation maps with significant clusters are shown and summarized in Fig. 3 and Table 3.

**Table 3:** Brain activation maps with significant clusters within the language mask.

Brain regions per sentence type	BA	Peak MNI		Voxels	*t*-value
Correct sentences					
Gram minus PC in the Syntactic Task					
Left superior + middle temporal gyri	22/21	− 62 − 12 6	2883		16.77
Left IFG pars triangularis	45	− 38 28 − 14	516		5.77
SCon minus PC in the Semantic Task					
Left superior + middle temporal gyri	22/21	− 62 16 24	3290		15.56
Left IFG pars opercularis + triangularis	44/45	− 50 16 24	1327		7.07
Left middle temporal gyrus	21	− 42 − 66 22	73		4.27
Syntactic Task (Gram minus PC) > Semantic Task (SCon minus PC)					
n.s.					
Semantic Task (SCon minus PC) > Syntactic Task (Gram minus PC)					
n.s.					
Conjunction of Both Tasks [(Gram minus PC) ∩ (SCon minus PC)]					
Left superior + middle temporal gyri	22/21	− 64 − 12 6	3117		16.69
Left IFG pars triangularis + opercularis	45/44	− 46 26 − 2	1091		6.15
Left middle temporal gyrus	21	− 48 − 60 − 4	64		4.12
Incorrect sentences					
FVio minus PC in the Syntactic Task					
Left superior + middle temporal gyri	22/21	− 62 − 14 6	2902		15.83
Left IFG pars triangularis	45	− 46 28 2	621		7.18
Left IFG pars opercularis	44	− 52 16 28	145		4.24
InCon minus PC in the Semantic Task					
Left superior + middle temporal gyri	22/21	− 66 − 28 8	2646		15.11
Left IFG pars triangularis	45	− 38 30 − 14	379		6.42
Left IFG pars opercularis	44	− 50 14 26	61		4.24
Syntactic Task (FVio minus PC) > Semantic Task (InCon minus PC)					
Left middle temporal gyrus + superior temporal sulcus	21/22	− 56 − 34 4	277		5.08
Semantic Task (InCon minus PC) > Syntactic Task (Fvio minus PC)					
n.s.					
Conjunction of Both Tasks [Fvio minus PC] ∩ (InCon minus PC)]					
Left superior + middle temporal gyri	22/21	− 66 − 28 8	2932		14.73
Left IFG pars triangularis + opercularis	45/44	− 38 28 − 14	718		5.90

n.s. suggests no significant clusters were observed.

### Multi-voxel pattern analysis

#### Correct sentences

**Fig. 4:**
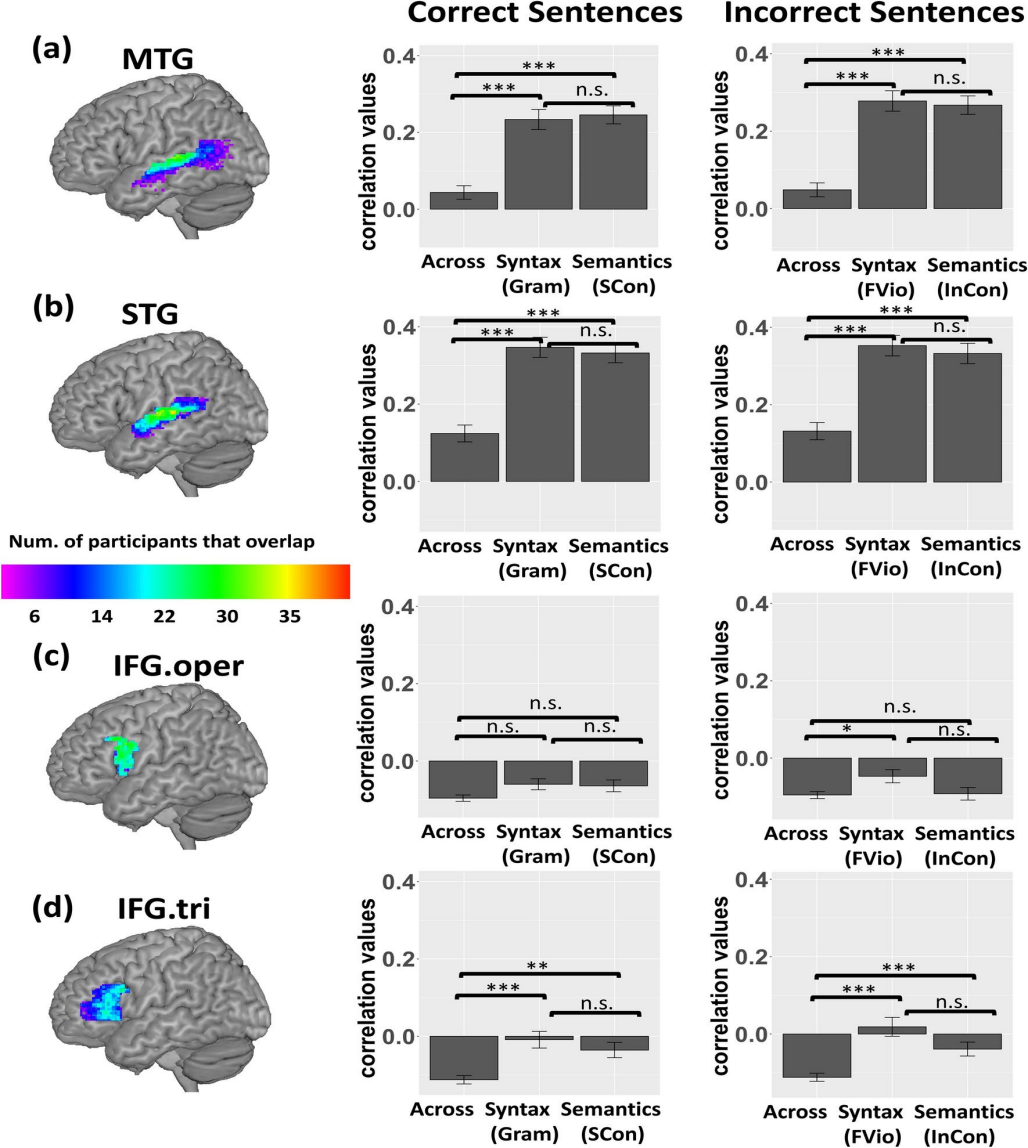
Statistics for the within-syntactic, the within-semantic, and the across-task correlations in (**a**) the left MTG, (**b**) the left STG, (**c**) the left IFG pars opercularis (IFG.oper), and (**d**) the left IFG pars triangularis (IFG.tri). The left column shows the overlap of individualized top 250 voxels across participants within each ROI. Color bar indicates the number of participants that overlapped. The middle column shows the group level comparisons among the within-semantic, within-syntactic, and across-task correlations for correct sentences. The right column shows the group level comparisons for incorrect sentences. Gram: grammatically correct condition; FVio: finiteness violated condition; Gram and FVio refer to the within-syntactic correlations of correct and incorrect sentences, respectively. SCon: strongly congruent condition; InCon: incongruent condition; SCon and InCon refer to the within-semantic correlations of correct and incorrect sentences, respectively. Across refers to the across-task correlations. *p < 0.05, **p < 0.01, ***p < 0.001, Bonferroni corrected. "n.s." means not significant. Error bar represents 1 standard error (SE) above and below the mean.

In the left MTG (see Fig. 4a), the within-Gram correlations were significantly higher than the across-task correlations [*t* (63) = 6.250, *p* < 0.001]. The within-SCon correlations were also significantly greater than the across-task correlations [*t* (63) = 7.635, *p* < 0.001]. There was no difference between the within-Gram and the within-SCon correlations [*t* (63) = − 0.433, *p* = 0.999, Bonferroni corrected]. This result suggests that the left MTG was sensitive to both semantic and syntactic information with no specialization during correct sentence processing.

In the left STG (see Fig. 4b), the within-Gram correlations were significantly higher than the across-task correlations [*t* (63) = 7.021, *p* < 0.001]. The within-SCon correlations were also significantly higher than the across-task correlations [*t* (63) = 7.189, *p* < 0.001]. There was no significant difference between the two within-task correlations [*t* (63) = 0.515, *p* = 0.999, Bonferroni corrected]. This result suggests that the left STG was sensitive to both semantic and syntactic information with no specialization during correct sentence processing.

In the left IFG pars opercularis (see Fig. 4c), there was no significant difference between the within-Gram correlations and the across-task correlations [*t* (63) = 2.191, *p* = 0.096, Bonferroni corrected]. There was also no significant difference between the within-SCon correlations and the across-task correlations [*t* (63) = 1.762, *p* = 0.249, Bonferroni corrected]. The two within-task correlations did not differ significantly [*t* (63) = 0.214, *p* = 0.999, Bonferroni corrected]. This result suggests that the left IFG pars opercularis was not sensitive to either semantic or syntactic information during correct sentence processing.

In the left IFG pars triangularis (see Fig. 4d), the within-Gram correlations were significantly higher than the across-task correlations [*t* (63) = 4.044, *p* < 0.001]. The within-SCon correlations were also significantly higher than the across-task correlations [*t* (63) = 3.524, *p* = 0.003, Bonferroni corrected]. There was no significant difference between the two within-task correlations [*t* (63) = 1.231, *p* = 0.669, Bonferroni corrected]. This result suggests that the left IFG pars triangularis was sensitive to both semantic and syntactic information with no specialization during correct sentence processing.

#### Incorrect sentences

In the left MTG (see Fig. 4a), the within-FVio correlations were significantly higher than the across-task correlations [*t* (63) = 8.014, *p* < 0.001]. The within-InCon correlations were also significantly greater than the across-task correlations [*t* (63) = 7.886, *p* < 0.001]. There was no difference between the within-FVio and the within-InCon correlations [*t* (63) = 0.368, *p* = 0.999, Bonferroni corrected]. This result suggests that the left MTG was sensitive to both semantic and syntactic information with no specialization during incorrect sentence processing.

In the left STG (see Fig. 4b), the within-FVio correlations were significantly higher than the across-task correlations [*t* (63) = 7.684, *p* < 0.001]. The within-InCon correlations were also significantly higher than the across-task correlations [*t* (63) = 6.505, *p* < 0.001]. There was no significant difference between the two within-task correlations [*t* (63) = 0.670, *p* = 0.999, Bonferroni corrected]. This result suggests that the left STG was sensitive to both semantic and syntactic information with no specialization during incorrect sentence processing.

In the left IFG pars opercularis (see Fig. 4c), the within-FVio correlations were significantly higher than the across-task correlations [*t* (63) = 2.794, *p* = 0.021, Bonferroni corrected]. However, there was no significant difference between the within-InCon correlations and the across-task correlations [*t* (63) = 0.181, *p* = 0.999, Bonferroni corrected]. The within-FVio correlations did not significantly differ from the within-InCon correlations [*t* (63) = 1.929, *p* = 0.174, Bonferroni corrected]. This result provides strong evidence for syntactic specialization in the left IFG pars opercularis for incorrect sentence processing.

In the left IFG pars triangularis (see Fig. 4d), the within-FVio correlations were significantly higher than the across-task correlations [*t* (63) = 5.211, *p* < 0.001]. The within-InCon correlations were also significantly higher than the across-task correlations [*t* (63) = 3.779, *p* < 0.001]. There was no significant difference between the two within-task correlations [*t* (63) = 2.370, *p* = 0.062, Bonferroni corrected]. This result suggests that the left IFG pars triangularis was sensitive to both semantic and syntactic information with no specialization during incorrect sentence processing.

## Discussion

The current study examined semantic and syntactic specialization during auditory sentence processing in 9- to 10-year-old children using both univariate analyses and MVPA. Like previous studies on younger children^26,27^, we did not find evidence for language specialization using univariate analysis. However, using MVPA, we observed syntactic specialization in the left IFG pars opercularis for incorrect sentence processing as evidenced by significantly higher within-syntactic (but not within-semantic) correlations than across-task correlations. In addition, we found that the left STG, the left MTG, and the left IFG triangularis were sensitive to both semantic and syntactic information with no evidence of specialization regardless of sentence correctness. This finding is supported by the findings of both within-task (within-syntactic and within-semantic) correlations being significantly higher than across-task correlations and no differences between the two within-task correlations. These findings, together with our previous work on younger children^26,27^, have implications for the neural development of semantic and syntactic specialization during sentence comprehension. Moreover, these findings shed light on the current debates about whether syntax is separable from semantic processing^16,18^.

### Syntactic specialization in the left IFG pars opercularis for incorrect sentence processing

One primary finding of the current study is that, in 9- to 10-year-old children, we observed significantly higher within-syntactic (for finiteness violated, FVio) but not within-semantic (for incongruous, InCon) correlations than across-task correlations for MVPA in the left IFG pars opercularis, suggesting that this region is specialized for syntactic information during incorrect sentence processing. Using the same experimental design and analytical approach, our previous studies on younger children ages 5-to-6 and 7-to-8 observed no sensitivity to either semantic or syntactic information in the IFG pars opercularis^26,27^. The syntactic specialization in the IFG pars opercularis observed only in 9- to 10-year-old children in the current study is consistent with a previous developmental study by Skeide et al.^23^. In their study, despite using different syntactic manipulations (i.e., object- versus subject -initial) and analytical approaches (i.e., univariate analyses), Skeide et al.^23^ also reported syntactic specialization in the left IFG pars opercularis in 9- to 10-year-old children but not in the younger (i.e., 3–4-year-old and 6–7-year-old) groups. Our finding of neural specialization of syntax only for the older children is consistent with behavioral research showing that morphosyntactic skills are mastered by about the age of 9 to 10 years old^24^. Our evidence of syntactic specialization is also supported by meta-analyses on adults^41–43^ reporting that the left IFG pars opercularis is one of the regions sensitive to syntactic information during sentence comprehension, such as word order (sentences > pure content/function word lists in Zaccarella et al.^41^; noncanonical > canonical sentences in Walenski et al.^42^), and syntactic complexity manipulations (Merge + Movement + Reanalysis in Heard and Lee^43^). Overall, together with our previous work on younger children^26,27^, the emergence of syntactic specialization in the pars opercularis observed only in 9- to 10-year-old children in the current study suggests a gradual maturation of frontal mechanisms for language comprehension, which is consistent with the neuro-developmental model proposed by Skeide and Friederici^15^.

In the current study, syntactic specialization in the left IFG pars opercularis was only observed for incorrect but not correct sentence processing. As compared to correct sentences, processing incorrect sentences requires more cognitive control processes while individuals are trying to make sense of them^34^. Literature has suggested that the left IFG is responsible for the detection and resolution of incompatible representations^44^. Therefore, our finding of syntactic specialization in the left IFG pars opercularis only for incorrect but not correct sentences was likely a result of increased syntactic evaluation and surprise for processing anomalous sentences. As can be observed in our MVPA results, both within-task and across-task correlations in the frontal regions were small and slightly negative (-0.1 < *r*s < 0). Although the meanings of these negative correlations are unknown, they have also been consistently observed in our previous studies on younger children^26,27^. Regardless, we observed that within-syntactic (but not within-semantic) correlations were significantly higher than across-task correlations in the left IFG pars opercularis in 9- to 10-year-old children, suggesting that this region can differentiate syntax from semantic information during incorrect sentence processing.

### The left STG sensitive to both semantic and syntactic information with no specialization, regardless of sentence correctness

Using MVPA, in the left STG during both correct and incorrect sentence processing, we observed that both within-semantic and within-syntactic correlations were significantly higher than across-task correlations with no significant difference between the two within-task correlations. These results suggest that brain activation patterns within the left STG were sensitive to both semantic and syntactic information. This finding was expected and has also been consistently observed in our previous studies on younger children^26,27^. Our finding of STG being sensitive to both semantic and syntactic information in developing children aligns with Friederici’s^16^ language comprehension model which argues the left STG contributes to the integration of both semantic and syntactic processes from other brain regions. However, given that we did not examine functional connectivity, it remains unclear whether the sensitivity to semantic and syntactic information observed in the left STG was driven by top-down processes from other regions, a question that needs future studies to address.

### The left MTG and IFG pars triangularis sensitive to both semantic and syntactic information with no specialization, regardless of the sentence correctness

Similar to the finding in the left STG, our MVPA results showed that the left MTG as well as the left IFG pars triangularis exhibited sensitivity to both semantic and syntactic information, regardless of sentence correctness. This finding was unexpected. Using the same experimental design and analytical approach, our previous studies showed semantic specialization in the left MTG in 5- to 6-year-old children^26^ and semantic specialization in both the left MTG and the left IFG pars triangularis in 7- to 8-year-old children^27^. According to the Interactive Specialization theory^4^, which argues that brain cortices exhibit increased specialization as children develop, we should observe stronger semantic specialization in the left MTG and the left IFG pars triangularis in 9- to 10-year-old children. However, we found no evidence of semantic specialization in either region in the current study.

**Fig. 5:**
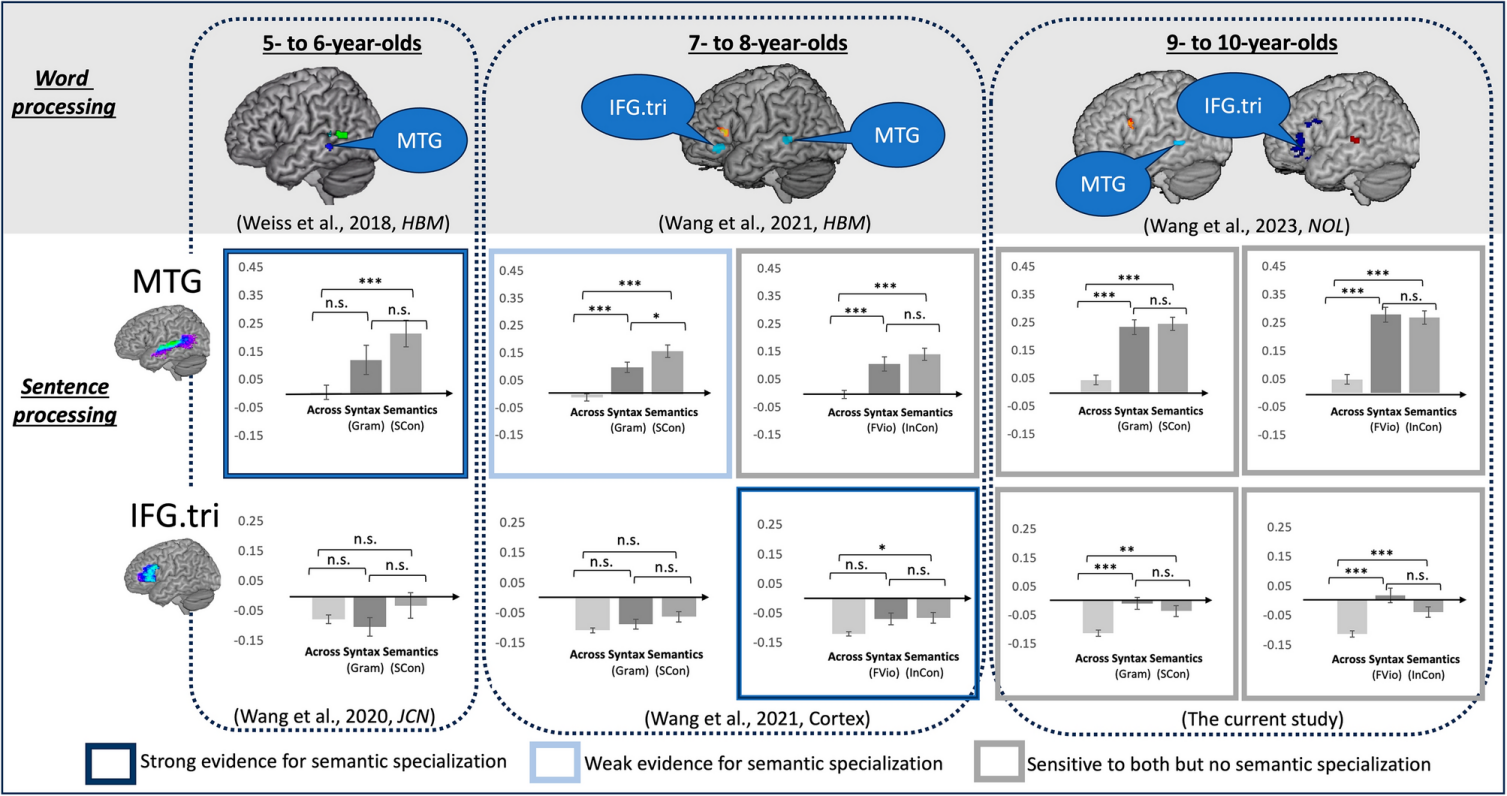
A summary of our previous and the current findings on semantic specialization in the left middle temporal gyrus (MTG) and inferior frontal gyrus pars triangularis (IFG.tri) in children at different ages for both word and sentence processing suggesting reduced semantic specialization. We hypothesize that children first rely on language regions that are semantically specialized at the word level during sentence processing. As children improve their syntactic skills, those semantic sensitive regions at the word-level also become more syntactically sensitive, leading to reduced semantic specialization. The upper figure shows studies during word processing that suggest a developmental transition of increased semantic specialization from the left MTG^12^ to both the left MTG and IFG pars triangularis^13,14^. The lower figure displays studies during sentence processing that indicate a developmental transition of reduced semantic specialization from strong^26^, to weak^27^, to none (the current study) in the left MTG, and from strong^25^ to none (the current study) in IFG pars triangularis.

One possible explanation for this unexpected finding is that during sentence processing children may first rely on language regions that are specialized for semantic processing at the word level. As children improve their syntactic skills^24^, those regions sensitive to semantics at the word-level also become more syntactically sensitive, leading to smaller difference when comparing semantic and syntactic processing during sentence comprehension. This argument is consistent with our previous studies in children ages 5-to-6, 7-to-8, and 9-to-10^12–14,26,27^. By directly comparing brain activity during a phonological and a semantic task during word-level processing, Weiss, Cweigenberg, and Booth^12^ found that semantic specialization only appeared in the left MTG in 5- to 6-year-old children. Correspondingly, our previous study^26^, which directly compared brain activity during a grammaticality and a plausibility task during sentence-level processing, showed that 5- to 6-year-old children demonstrated strong semantic specialization in the left MTG. Wang, Yamasaki, Weiss, and Booth^13^ used the same experimental design and analytical approach as in the Weiss et al.^12^ study of word-level processing and found that in addition to a semantic specialization effect in the left MTG, 7- to 8-year-old children began to show semantic specialization in the left IFG pars triangularis/orbitalis. In parallel, our previous study^27^ on sentence-level processing observed that 7- to 8-year-old children showed strong semantic specialization in the left IFG pars triangularis. In contrast to strong semantic specialization in the left IFG pars triangularis, this previous study^27^ only found weak semantic specialization in the left MTG. Thus, there seems to be weakened semantic specialization during sentence processing as children develop. Consistent with this developmental reduction in semantic specialization, in the current study, we observed that 9- to 10-year-old children did not show evidence of semantic specialization in either the left MTG or the left IFG pars triangularis, even though 9- to 10-year-olds continued to show semantic specialization in both areas during word-level processing^14^. Figure 5 shows a summary of our previous and the current findings on semantic specialization in the left MTG and IFG pars triangularis in children at different ages for both word and sentence processing.

This apparent reduced semantic specialization for sentence processing is not contradictory to the Interactive Specialization theory^4^ as the weakened specialization for semantic processing was due to increased sensitivity to syntactic information in the left MTG and the left IFG pars triangularis. In fact, we observed that the sensitivity to semantic representations in the left MTG and IFG pars triangularis either maintained or increased with development. Specifically, the mean difference between within-semantic and across-task correlations in the left MTG is 0.21 (*p* < 0.0001) for 5- to 6-year-olds, 0.17 (*p* < 0.0001) for 7- to 8-year-olds, and 0.20 (*p* < 0.0001) for 9- to 10-year-olds across studies^26,27^, suggesting that the left MTG is consistently sensitive to semantic information across development. In addition, the mean difference between the within-semantic and across-task correlations in the left IFG pars triangularis is 0.05 (*p* = 1) for 5- to 6-year-olds, 0.04 (*p* = 0.069) for 7- to 8-year-olds, and 0.08 (*p* < 0.0001) for 9- to 10-year-old children across studies^26,27^, suggesting that the left IFG pars triangularis increases in semantic sensitivity with development. Our series of fMRI studies aligns with several previous developmental studies using multiple neuroimaging modalities (e.g., fMRI in Wu et al.^22^; ERPs in Schneider et al.^45^; and Strotseva-Feinschmidt et al.^46^). In their studies, researchers have consistently argued that syntactic processing relies more on semantic processing in early childhood. In summary, while there appears to be reduced semantic specialization in the left MTG and the left IFG pars triangularis during sentence comprehension, children’s brains exhibit a sustained or even heightened sensitivity to semantic information.

### Implications for the current debates in language theories

The first debate in the literature focuses on whether syntax is separable from semantic processing in the brain^16–19^. The current study in 9- to 10-year-old children, using MVPA but not univariate analyses, observed that within-semantic and within-syntactic correlations were larger than across-task correlations in the left STG, the left MTG, and the left IFG pars triangularis. Thus, the separation of semantic and syntactic processing was demonstrated only in shared brain regions rather than different activation amplitude at different brain areas. This finding supports the separation of semantic and syntactic processing in the brain and may partially explain why others have not observed syntactic specialization using univariate analyses (e.g., Fedorenko et al.^18^). In addition, our finding is consistent with a prior study by Murphy et al.^50^ using intracranial recording, which revealed a mosaic located at the lower bank of the posterior superior temporal sulcus (pSTS), in which closely neighboring cortical sites displayed exclusive sensitivity to either lexicality (semantics) or phrase structure (syntax), but not both.

The second debate is related to where syntactic processing locates in the brain^16,17,19^. In Friederici’s^16^ model, the left IFG pars opercularis is hypothesized to be the core region for syntactic processing, whereas Hagoort^17^ proposed that the left IFG pars triangularis and MTG are specialized for syntactic processing. Our study on 9- to 10-year-old children suggests that the left IFG pars opercularis is specialized for syntactic processing, which is supportive of Friederici’s^16^ model. However, the syntactic specialization effect was only observed for incorrect but not correct sentences, suggesting that the left IFG pars opercularis may be involved only when the increased effort of syntactic evaluation is needed. Given that semantic processing in the IFG pars triangularis exhibited a developmental transition from incorrect to correct sentences in children from ages 7-to-8 to ages 9-to-10 (see Fig. 5), we speculate that syntactic processing in the left IFG pars opercularis may also change as children grow older. Therefore, before reaching a conclusion on whether the left IFG pars opercularis is a core region for syntactic processing as suggested by Friederici^16,47^, future studies using the same experimental design and analytical approaches on older children or adults are needed. Those studies on adults will be crucial in determining the eventual status of the neural specialization of syntactic and semantic processing. Friederici^16^ also proposed that the left STG plays a role in integrating both semantic and syntactic information, and similarly, but adopting a lexicalized view of syntax, Matchin and Hickok^19^ suggests that semantic and syntactic processing are intertwined in the left IFG pars triangularis and MTG. We found that the left IFG pars triangularis, the left MTG, and the left STG exhibited sensitivity to both semantic and syntactic information irrespective of sentence correctness, without showing indications of specialization. This intertwined semantic and syntactic processing in shared language regions observed is supportive of the lexicalized view of syntax. Consistent with this argument, recent fMRI studies on adults^48,49^, using an experimental design (i.e., contrasting sentences, Jabberwocky sentences, word lists, and nonword lists) or natural language processing approach, observed that most brain regions involved in language were sensitive to both syntactic and semantic variables. Using intracranial recording on adult patients, a prior study by Murphy et al.^50^ suggests that the pSTS encodes the minimal syntactic structure (i.e., the phrase structure), while the IFG pars triangularis is associated with anticipation. Therefore, temporal language areas may be more engaged in the initial generation of syntax, whereas frontal language regions are responsible for higher-level syntactic anticipation and evaluation. Our current study with 9- to 10-year-old children revealed neural sensitivity to syntax in sentence processing in all language regions, including the left IFG pars opercularis, the left IFG pars triangularis, the left STG, and the left MTG. Across our series of fMRI studies involving children aged 5, 7, and 9, more frontal sites exhibited more sensitivity to syntactic information as children matured (see Fig. 5), suggesting a developmental progression of syntactic processing from basic encoding to higher-level evaluation.

### Conclusion

In the current study, we observed that 9- to 10-year-old children showed syntactic specialization in the left IFG pars opercularis for incorrect sentence processing, which is consistent with the argument by Friederici^16,45^ that this area is key for syntactic processing. We also observed that the left STG, the left MTG, and the left IFG pars triangularis were sensitive to both semantic and syntactic information, with no evidence of specialization, supporting other theoretical models that take a lexicalized view of syntax^19^. Compared to our previous studies on younger children using the same experimental design and analytical approach^24,25^, the current study suggests an emergence of syntactic specialization in the IFG pars opercularis at 9 to 10 years old. Additionally, as children age from 5 to 10 years old, there appears to be reduced semantic specialization accompanied by an increased sensitivity to syntactic information in the left MTG and IFG pars triangularis.

## Supplementary Information

Supplementary Information.

## Data Availability

The specific subjects and the scripts used for this current study are shared on Github https://github.com/wangjinvandy/Syntactic_Semantic_Specialization_9_10_yo. The original data is on OpenNeuro https://openneuro.org/datasets/ds003604.
