# Supplementary material for: Syntactic and semantic specialization in 9- to 10-year-old children during auditory sentence processing
Source: Sci Rep. 2024 Nov 6;14:26965. doi: 10.1038/s41598-024-76907-8 (PMC11541780; doi:10.1038/s41598-024-76907-8)
Supplement: Supplementary file 1 — Supplementary Information. [file 41598_2024_76907_MOESM1_ESM.docx]

**Syntactic and semantic specialization in 9- to 10-year-old children during auditory sentence processing**

Jin Wang^a^, Neelima Wagley^b^, Mabel Rice^c^, Nadine Gaab^d^, James R Booth^e^

^a^ School of Education and Information Studies, University of California, Los Angeles, CA. USA.

^b^ Speech and Hearing Sciences, Arizona State University, Tempe, AZ. USA.

^c^ Child Language Doctoral Program, University of Kansas, Lawrence, KS. USA.

^d^ Graduate School of Education, Harvard University, Cambridge, MA. USA.

^e^ Department of Psychology and Human Development, Vanderbilt University, Nashville, TN. USA.

Supplementary Material

In the left MTG (see **Figure 1S, upper left**), the within-PVio correlations were significantly higher than the across-task correlations [*t* (63) = 6.620, *p* < .001]. The within-InCon correlations were also significantly greater than the across-task correlations [*t* (63) = 8.499, *p* < .001]. There was no difference between the within-FVio and the within-InCon correlations [*t* (63) = -0.923, *p* = .359]. This result suggests that the left MTG was sensitive to both semantic and syntactic information with no specialization.

In the left STG (see **Figure 1S, upper right**), the within-PVio correlations were significantly higher than the across-task correlations [*t* (63) = 8.181, *p* < .001]. The within-InCon correlations were also significantly higher than the across-task correlations [*t* (63) = 6.963, *p* < .001]. There was no significant difference between the two within-task correlations [*t* (63) = .555, *p* = .581]. This result suggests that the left STG was sensitive to both semantic and syntactic information with no specialization.

In the left IFG pars opercularis (see **Figure 1S, lower left**), because the within-PVio correlations were not normally distributed (Shapiro test p <.05), Wilcoxon Signed Rank test was conducted. We observed a trend of higher within-PVio correlations than the across-task correlations [*V* = 1280, *p* = .055]. In addition, there was no significant difference between the within-InCon correlations and the across-task correlations [*t* (63) = 0.098, *p* = .461]. The within-PVio correlations did not significantly differ from the within-InCon correlations [*V* = 1223, *p* = .222]. This result indicates a trend for syntactic specialization in the left IFG pars opercularis.

In the left IFG pars triangularis (see **Figure 1S, lower right**), because the within-PVio correlations were not normally distributed (Shapiro test p <.05), Wilcoxon Signed Rank test was conducted. We observed that the within-PVio correlations were significantly higher than the across-task correlations [*V* = 1620, *p* < .001]. The within-InCon correlations were also significantly higher than the across-task correlations [*t* (63) = 3.293, *p* < .001]. There was no significant difference between the two within-task correlations [*V = 1229*, *p* = .208]. This result suggests that the left IFG pars triangularis was sensitive to both semantic and syntactic information with no specialization.


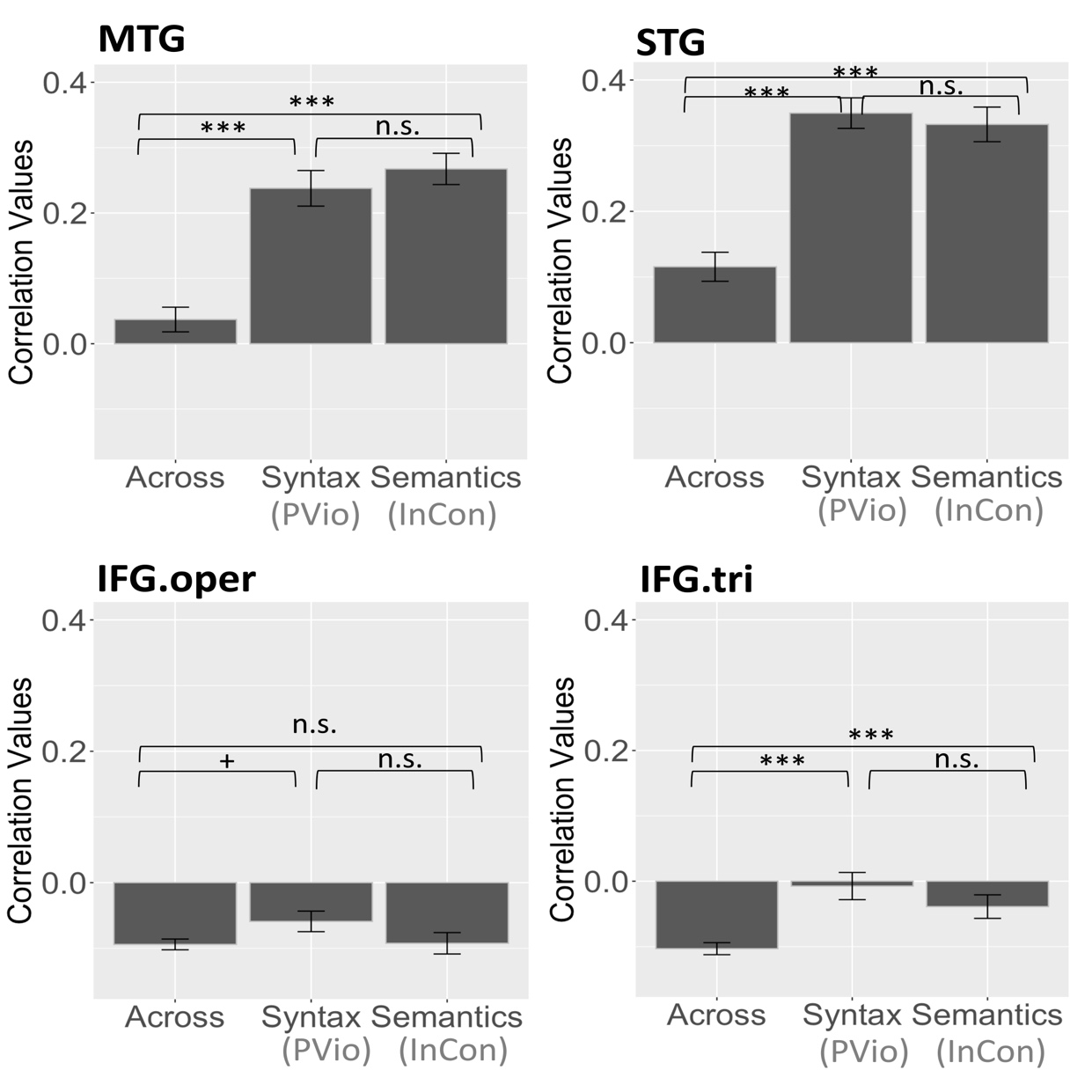


Figure 1S. Statistics for the within-syntactic, the within-semantic, and the across-task correlations in the left MTG, the left STG, the left IFG pars opercularis (IFG.oper), and the left IFG pars triangularis (IFG.tri). Across: the across-task correlations. PVio: Plurality violated condition in the Grammaticality Task tapping into syntax. InCon: Incongruent condition in the Plausibility Task tapping into semantics. + indicates p < .10 and *** indicates *p* < .001. "n.s." = not significant. The error bar represents 1 standard error (SE) above and below the mean.
